# Sunlight exposed body surface area is associated with serum 25-hydroxyvitamin D (25(OH)D) level in pregnant Minangkabau women, Indonesia

**DOI:** 10.1186/s40795-020-00342-x

**Published:** 2020-05-18

**Authors:** Cimi Ilmiawati, Athica Oviana, Andi Friadi, Mohamad Reza

**Affiliations:** 1 Department of Pharmacology, Faculty of Medicine, Andalas University, Gedung A, Lantai 1, Main Campus Limau Manis, Pauh, PO. BOX 49, Padang, West Sumatra 25166 Indonesia; 2 Graduate Program of Midwifery, Faculty of Medicine, Andalas University, Padang, West Sumatra Indonesia; 3 School of Midwifery, STIKes Perintis, Padang, West Sumatra Indonesia; 4 Department of Obstetrics and Gynecology, Faculty of Medicine, Andalas University, Padang, West Sumatra Indonesia; 5 Department of Biology, Faculty of Medicine, Andalas University, Padang, West Sumatra Indonesia

**Keywords:** Body surface area, Pregnant, Sunlight exposure, Vitamin D

## Abstract

**Background:**

Vitamin D deficiency is highly prevalent in women, and living in a tropical country with a year-round abundance of sunlight as the primary source of vitamin D does not seem to guarantee adequate serum 25(OH)D. While living in the tropics, Minangkabau women are known to dress specifically according to their culture. This study was aimed to elucidate the association of sunlight exposed body surface area with serum 25(OH)D in pregnant Minangkabau women of Indonesia.

**Methods:**

We performed a cross-sectional study on 88 Minangkabau women in late pregnancy. Lifestyle data were collected using a questionnaire, and dietary intake of vitamin D was calculated from 24-h food recall. The skin pigmentation type was determined by the Fitzpatrick scale, and the body surface area exposed to sunlight was estimated. Serum 25(OH)D was quantified by ELISA method. Serum 25(OH)D differences according to the duration of sunlight exposure, skin pigmentation type, and sunscreen use were statistically analyzed by ANOVA. The correlation of sunlight exposed body surface area and serum 25(OH)D was analyzed by Spearman’s correlation.

**Results:**

Nearly half of the subjects (*n* = 40; 45.5%) were deficient in vitamin D (< 20 ng/ml) with serum 25(OH)D level 23.0 ± 10.0 ng/ml (mean ± SD) and the estimated daily intake of vitamin D was 5.6 ± 3.9 μg/1000 kcal/day (mean ± SD). The median percentage of body area exposed to sunlight was 15.8%. There were no differences in serum 25(OH)D levels according to sunlight exposure time, skin pigmentation type, and sunscreen use. The percentage of body area exposed to sunlight was positively correlated with serum 25(OH)D level (Spearman’s *ρ* = 0.403; *p* < 0.001).

**Conclusions:**

Vitamin D deficiency is prevalent in pregnant Minangkabau women. Since increasing body surface area exposed to sunlight may not be culturally acceptable, vitamin D supplementation needs to be considered in this population.

## Background

Vitamin D deficiency (VDD) has been reported from many countries and across different populations, making it a global public health concern [1]. In South Asia, the prevalence of VDD was estimated to be 70% or more, while in South-East Asia, it varied between 6 and 70% [2]. Currently, there is no scientific agreement regarding the cut-off points to define low vitamin D status due to non-standardized laboratory methods to measure the biomarker, total serum 25-hydroxyvitamin D [25(OH)D]. Serum 25(OH)D value below 12 ng/mL (30 nmol/L) is considered to be associated with elevated risk of osteomalacia, whereas value between 20 and 50 ng/mL (50–125 nmol/L) appeared to be sufficient for the skeletal health in the general population [3] and a blood level above 30 ng/mL to optimize vitamin D’s effect on calcium metabolism [4]. In China, it was reported that 63.7% of pregnant women were vitamin D deficient [25(OH)D < 50 nmol/L] [5], while in Turkey, the number was 94.8% [6]. In Vietnam, 60% of women in late pregnancy had low serum 25(OH)D level (< 75 nmol/L) [7]. A study in Kenya, a tropical country, showed that 51% of women had insufficient (< 75 nmol/L), and 21% had deficient serum 25(OH)D level (< 50 nmol/L) [8]. With the high prevalence of VDD in women reported in sunny countries [8–10], VDD in pregnant women needs particular attention because of the potential unwanted birth outcome.

VDD during pregnancy may adversely affect the offspring. Monitoring the serum level of 25(OH)D during the antenatal period is warranted as a preventive measure to decrease morbidity during pregnancy and lactation period and to mitigate the adverse effect on the fetus, newborn, and child [11]. Studies in Poland and the US found that VDD in pregnant women with serum 25(OH)D < 75 nmol/L was associated with an increased risk of preeclampsia [12, 13]. Lower serum 25(OH)D has been shown to associate with increased risk of macrosomia, and vitamin D supplementation is advised during pregnancy [14]. A study in Kenya showed that vitamin D insufficiency is associated with neonatal stunting [8]. Identifying factors related to serum 25(OH)D in a specific population is essential to devise appropriate measures to prevent deficiency.

Geographical factor, like season [15] and latitude [16], affects the intensity of ultraviolet B (UVB) radiation as the primary source of vitamin D3 synthesis in the exposed skin [17]. Other factors such as skin pigmentation type [18], amount of sunlight exposed body surface area (BSA) [19], sunscreen application, and dressing [20] may also influence serum 25(OH)D level. Daily dressing habits may affect serum 25(OH)D level because the type of fabric used may prevent UVB radiation absorption by the skin. Moreover, the size of the body surface area exposed to sunlight determines the amount of vitamin D synthesis in the skin [20].

Minangkabau women living in West Sumatra, Indonesia, are known to adhere to culturally acceptable dressing code in their daily life. It is common to cover the whole-body area except for the face, hands, and feet. A recent study in young Minangkabau women found that 97% of subjects were vitamin D deficient and that sleep quality, dietary intake, and sunscreen use were predictors of serum 25(OH)D [21]. The research underlines that residing in a tropical country does not guarantee the adequacy of serum vitamin D, and other factors need to be considered. Considering the highly prevalent low 25(OH)D serum in Indonesian women, the body-covering dressing characteristic of Minangkabau women, and the limited study on pregnant women, we undertook research examining the association of sunlight exposed body surface area with serum 25(OH)D level in Minangkabau women during late pregnancy.

## Methods

### Study subjects

This study was approved by the ethics committee of the Faculty of Medicine Andalas University (Approval No.005/KEP/FK/2019). Eighty-eight last trimester healthy pregnant women were recruited from February to June 2019 from a public health center in Padang by purposive sampling.

### Dietary vitamin D intake assessment

Subjects were interviewed by using a structured questionnaire, and 24-h food recall for the last 2 days (one weekday and one weekend) was employed to assess vitamin D intake [22]. The 24-h food recall method was performed as a direct interview by a trained interviewer and was facilitated with photographs of various serving sizes of foods. Dietary vitamin D intake was calculated by using the Nutrisurvey software with the Indonesian food database [23].

### Sunlight exposure, sunscreen use, and skin pigmentation type assessment

Questionnaire-guided interview was performed to obtain data on duration of sunlight exposure during the last 2 days (in minute) [24], on habitual dressing when going outdoor (percentage of body surface area exposed to sunlight) [19], on skin pigmentation (Fitzpatrick’s scale) [25], and sunscreen application (regular, irregular, non-user) [20]. The skin pigmentation type was also assessed by observation [26].

### Serum 25(OH)D analysis

All enrolled pregnant women had blood samples taken from their antecubital vein. Blood samples were directly transferred and stored in the biomedical laboratory at the Faculty of Medicine, Andalas University, for serum 25(OH)D assay. The serum samples were separated by centrifugation at 3500 rpm at 4 °C for 10 min, then stored in aliquots at − 80 °C. Serum 25(OH)D level was measured by enzyme-linked immunosorbent assay (ELISA) method by using 25(OH)D ELISA Kit (Can-VD-510) produced by Diagnostic Biochem Canada (DBC®) [9, 27]. Vitamin D status in this study was defined using the cut-off points suggested by Grant & Holick where vitamin D levels categorized as deficient (< 50 nmol/L = < 20 ng/mL), insufficient (50–79 nmol/L = 20–31 ng/mL) or sufficient (80–250 nmol/L = 32–100 ng/mL) [28].

### Statistical analysis

Statistical analysis was performed using parametric tests (Pearson correlation and One-Way ANOVA) on normally distributed data. Data with non-normal distribution were logarithmically transformed (*log*10) to approximate a normal distribution. A non-parametric test (Spearman correlation) was performed on data with non-normal distribution. Statistical significance was determined at a *p*-value of less than 0.05. All data were analyzed using IBM SPSS version 20.0.

## Results

Our findings showed that serum 25(OH)D level in third-trimester pregnant Minangkabau women was 23.0 ± 10.0 ng/ml (mean ± SD) and estimated daily intake of vitamin D 5.6 ± 3.9 μg/1000 kcal/day (mean ± SD). Nearly a third (29.5%) of the women were exposed to sunlight < 30 min/day and the median BSA exposed to sunlight was 15.8%. Most of the subjects were of type V skin pigmentation (86.4%) and were non-sunscreen users (79.5%). Other characteristics of subjects are presented in Table 1. We have previously reported that the dietary vitamin D intake in our subjects was not statistically significantly correlated with their levels of serum 25(OH)D [29].

**Table 1:** Characteristics of pregnant Minangkabau women in late pregnancy (*n* = 88)

Characteristic	*f*	%	Mean	SD	Min	Max	Median
**Age (year)**			30.6	5:03	17	41	30.5
**Gestational age (week)**			32.6	3.7	28	40	32
**Number of pregnancy**			2.6	1.1	1	5	3
**Upper arm circumference (cm)**			27.6	3.9	20	38	27
**Skinfold thickness (cm)**			15.2	7.2	3	35	12
**Serum 25(OH)D level (ng/mL)**			23.0	10.0	7.4	51.5	21.1
Deficiency (< 20 ng/mL)	40	45.5					
Insufficiency (20–31 ng/mL)	32	36.4					
Sufficiency (32–100 ng/mL)	16	18.2					
**Vitamin D intake (μg/1000 kcal/day)**			5.6	3.9	0.0	16.4	4.6
**Sunlight exposed BSA (%)**			15.2	6.8	4.9	28.4	15.8
**Duration of sunlight exposure (minute/day)**			110.8	156.7	0:00	990	60
< 30	26	29.5					
30–60	18	20.5					
> 60–120	25	28.4					
> 120	19	21.6					
**Skin pigmentation type (Fitzpatrick’s scale)**							
III	1	1.1					
IV	11	76					
V	76	86.4					
**Sunscreen use**							
Regular	7	8					
Irregular	11	12.5					
Non-user	70	79.5					

**Fig. 1:**
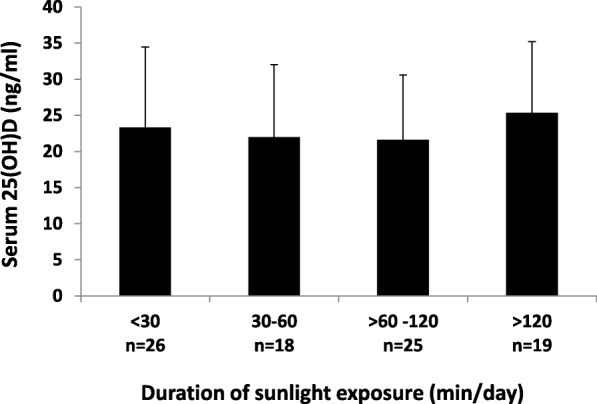
Serum 25(OH)D level (ng/ml) of pregnant Minangkabau women in late pregnancy according to the duration of sunlight exposure (min/day). No statistically significant difference in serum 25(OH)D between groups (*p* = 0.63; One-Way ANOVA)

**Fig. 2:**
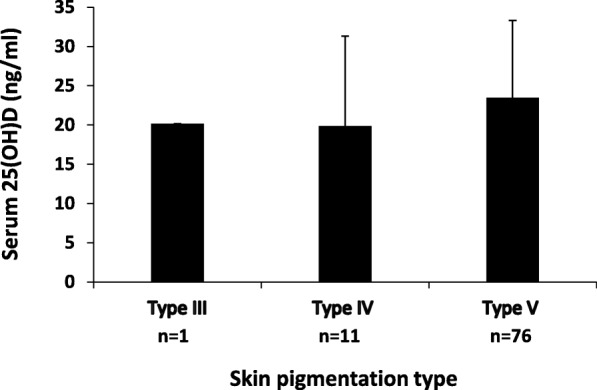
Serum 25(OH)D level (ng/ml) of pregnant Minangkabau women in late pregnancy according to their skin pigmentation type. No subject was categorized into skin pigmentation type I, II or VI. No statistically significant difference in serum 25(OH)D between groups (*p* = 0.51; One-Way ANOVA)

**Fig. 3:**
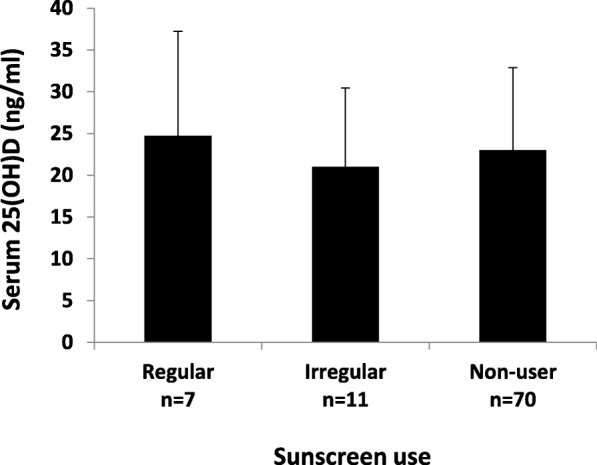
Serum 25(OH)D level (ng/ml) of pregnant Minangkabau women in late pregnancy according to their sunscreen use. No statistically significant difference in serum 25(OH)D between groups (*p* = 0.72; One-Way ANOVA)

Comparisons were performed to examine whether the level of serum 25(OH)D in our subjects was different according to the duration of sunlight exposure, skin pigmentation type, and sunscreen use. The results showed that there was no statistically significant difference in serum 25(OH)D level according to the duration of sunlight exposure (< 30, 30–60, > 60–120, and > 120 min/day; One-Way ANOVA; *p* = 0.63; Fig. 1). Serum 25(OH)D level was also no difference in regards to skin pigmentation type (type III, IV, and V; One-Way ANOVA; *p* = 0.51, Fig. 2), and sunscreen use (regular, irregular, and non-user; One-Way ANOVA; *p* = 0.72, Fig. 3).

**Fig. 4:**
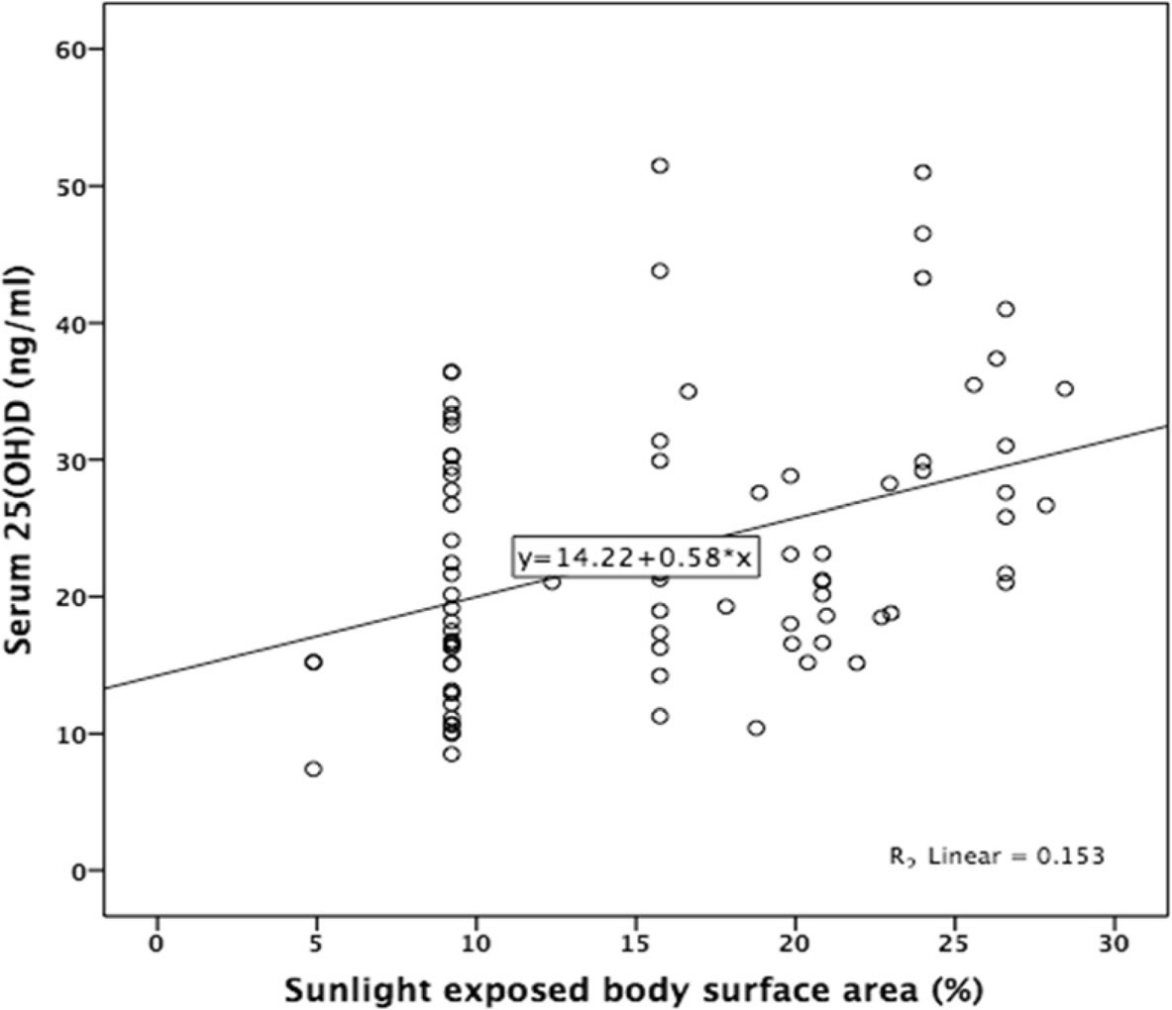
Correlation of sunlight exposed body surface area (%) with serum 25(OH)D level (ng/ml) in Minangkabau women of late pregnancy (Spearman’s rho = 0.403; *p* = < 0.001)

To examine the correlation between serum 25(OH)D level with sunlight exposed BSA, Spearman’s correlation test was performed, and the results showed that there was a statistically significant positive linear correlation between serum 25(OH)D level and percentage of BSA exposed to sunlight (R^2^ linear = 0.153; Spearman’s *ρ* = 0.403; *p* = < 0.001; Fig. 4).

## Discussion

Recent studies showed that VDD is common in Indonesian women [9, 27, 30]. Our previous study in healthy young Minangkabau women found that 97.5% of subjects had VDD [27]. In line with the finding earlier, in this study, we find that 81.8% of pregnant women have insufficient/deficient serum 25(OH)D level. Serum 25(OH)D level in our study (23.0 ± 10.0 ng/ml; mean ± SD) is similar to those of pregnant women in other areas in West Sumatra (25.4 ng/ml) [30] but higher than those of young Minangkabau women (median 10.5 ng/ml) [27].

In this study, almost all subjects were categorized into type V skin pigmentation according to Fitzpatrick’s scale, with few women had type III and type IV (Fig. 2). There were no pregnant women with type I, type II, or type VI [25] skin pigmentation as these women are of Minangkabau ethnic group having generally light brown to brown skin. Skin pigmentation largely depends on the concentration and type of melanin in epidermal keratinocytes. Melanin absorbs and scatters ultraviolet radiation, including UVB, therefore negatively affecting the conversion of 7-dehydrocholesterol in the skin into pre-vitamin D3. Studies have shown that people with darker skin had less effective photoproduction and, thus, lower levels of 25(OH)D [31, 32]. However, another study found no difference in serum 25(OH)D in dark-skinned (type V-VI of Fitzpatrick’s scale) versus fair-skinned (type I-IV of Fitzpatrick scale) subjects after UVB exposure [33]. In line with the previous finding, we did not find a difference in serum 25(OH)D level among type III, type IV, and type V skin type. Nevertheless, there is an uneven distribution of the number of subjects in each skin type, which may limit the interpretation of our result. Furthermore, skin pigmentation per se may have limited influence on serum 25(OH)D, and other physiological and environmental contexts, such as the percentage of sun-exposed BSA, play a determining role.

VDD in pregnancy is of particular importance because it may adversely impact the health of the newborn [8]. Minangkabau women living in West Sumatra is known to dress according to their culture where only the face, hands, and occasionally feet are exposed to sunlight. As UVB radiation from sunlight plays a central role in the dermal synthesis of vitamin D from 7-dehydrocholesterol, the dressing habit that hinders sunlight exposure may affect serum vitamin D level [20]. Our results support this notion where we found that the percentage of BSA exposed to sunlight correlates with serum 25(OH)D level, where 15.3% of serum 25(OH)D variability is explained by the percentage of skin exposed to sunlight. Our result also in accordance with the finding of a cross-sectional study in Ethiopia where sunlight exposed BSA is a predictor of serum 25(OH)D level [34]. However, our results are different from a study in healthy young Minangkabau women where no statistically significant correlation was found between sunlight exposed BSA and serum 25(OH)D [21]. It is possible that the contrast between subjects was not enough in that study, where 98% of the subjects wore a hijab (whole body-covering garment) with median sunlight exposed BSA was 7.8%. In the current study, not all of the women wore a hijab when going outdoor, and some chose to wear a sleeveless dress, creating contrast in data sufficient to detect the correlation between the degree of sunlight exposed BSA and serum 25(OH)D level.

As most of the subjects in our study wore a hijab, only around 15% of their body surface area was exposed to sunlight. Characteristics of fabrics used for the garment, such as colors, thickness, and weaving mode, may affect the garment’s effectivity in blocking UVB absorption by the skin [35]. Dark fabrics are twice as effective in absorbing UVB radiation compared to white ones. There is a significant difference in vitamin D synthesis between whole-body exposure compared to face-hands-feet only exposure to sunlight [20]. Sunlight UVB stimulates vitamin D synthesis from 7-dehydrocholesterol in the skin, where it will be stored in adipose tissue or be hydroxylated in the liver into 25(OH)D and undergoes further hydroxylation in the kidney to form the active calcitriol [36].

It has been proposed that skin exposure to sunlight for 7–25 min from 10 am to 3 pm, at least twice a week, where the face, arms, and legs are exposed without sunscreen application should be adequate to induce vitamin D synthesis [37, 38]. However, considering skin exposure is culturally inappropriate in Minangkabau ethnicity, sound advice would be for health care workers to monitor serum 25(OH)D level in pregnant women. With the highly prevalent VDD in women living in a tropical country, women should be provided with vitamin D supplementation before, during, after pregnancy.

Our research has several limitations. The estimation of dietary intake, sunlight exposure, and sunscreen use are approximations. Nevertheless, our findings are in line with previous studies in Indonesia [9, 10], strengthening the epidemiological evidence that VDD is prevalent in Indonesian women, and the lack of sunlight exposure is an essential determinant for VDD.

## Conclusions

Vitamin D deficiency is prevalent in late pregnancy. Since increasing skin exposure to sunlight is not culturally acceptable, pregnant Minangkabau women should be supplemented with vitamin D to mitigate this public health concern.

## Data Availability

Data from this study are available upon reasonable request to the corresponding author.

## Abbreviations

BSA: Body surface area
ELISA: Enzyme-linked immunosorbent assay
UVB: Ultraviolet B
VDD: Vitamin D deficiency
25(OH)D: 25-hydroxyvitamin D
